# Supplementary figures and images for: A Non-functional γ-Aminobutyric Acid Shunt Pathway in Cyanobacterium Synechocystis sp. PCC 6803 Enhances δ-Aminolevulinic Acid Accumulation under Modified Nutrient Conditions
Source: Int J Mol Sci. 2023 Jan 7;24(2):1213. doi: 10.3390/ijms24021213 (PMC9864891; doi:10.3390/ijms24021213)

X

X

X

X

X

X

X

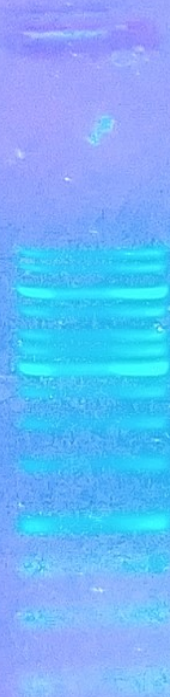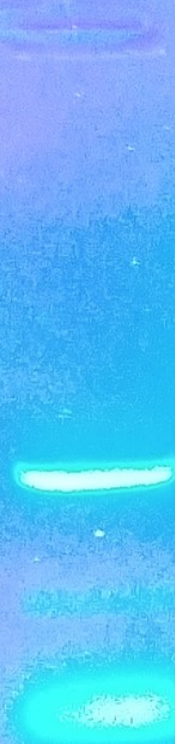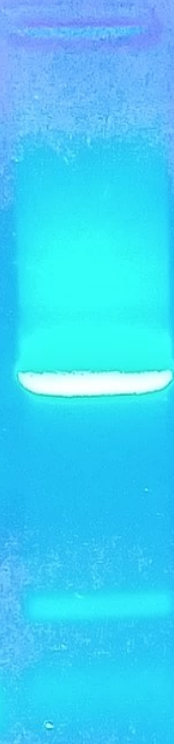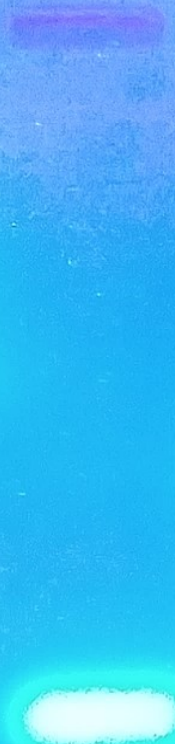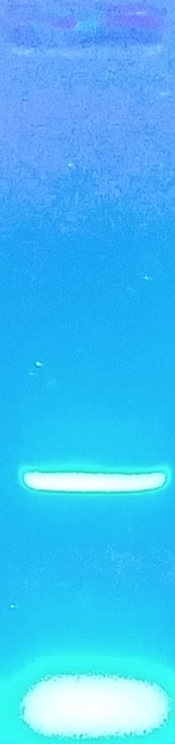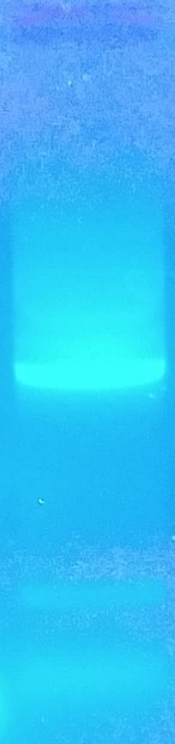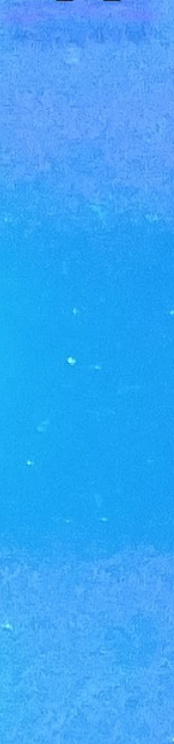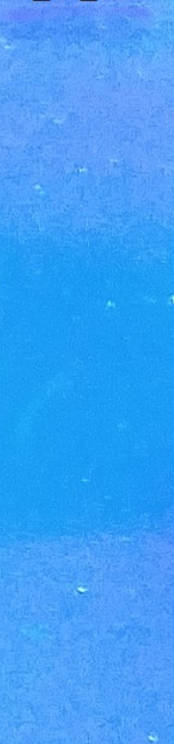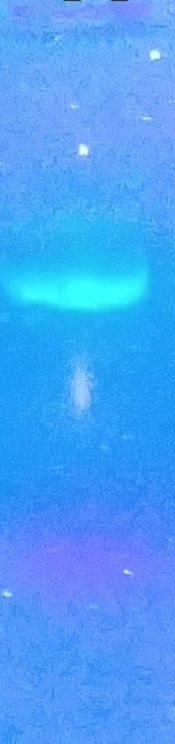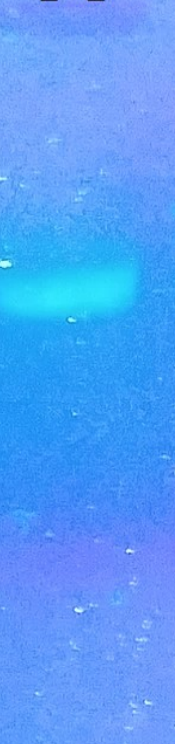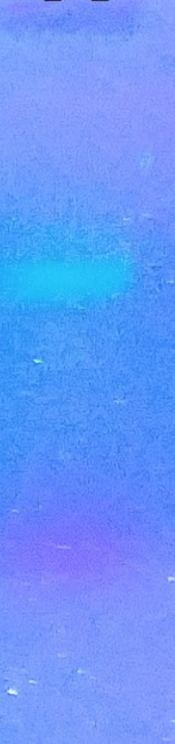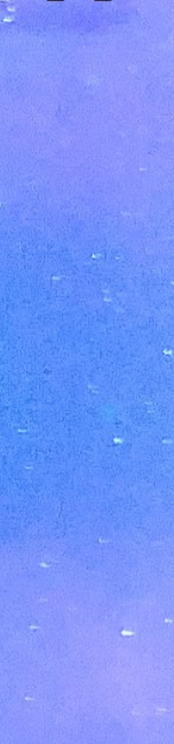

Supplement: Supplementary file 1 [file ijms-24-01213-s001.zip › ijms-2051526-supplementary.pdf]
